# Aging, inhibition and GABA

**DOI:** 10.18632/aging.101696

**Published:** 2018-12-05

**Authors:** Lisa Pauwels, Celine Maes, Stephan P. Swinnen

**Affiliations:** 1 KU Leuven, Movement Control and Neuroplasticity Research Group, Department of Movement Sciences, Group Biomedical Sciences, 3001 Leuven, Belgium; 2 Leuven Brain Institute (LBI), KU Leuven, Belgium

**Keywords:** aging, motor inhibition, Gamma-aminobutyric acid (GABA)

The concept of inhibition is deeply rooted in our society. This is not only evident in our day-to-day activities but also in our scientific endeavors to reveal its underlying mechanisms. Indeed, inhibition is expressed in various functional domains such as cognition, action and emotion. Being able to suppress preferred, addictive or intended behaviors is at the heart of our well-being and how we succeed in society. There is no single construct that fully captures the different expressions of inhibition. Here we focus on motor inhibition, defined as the ability to successfully cancel a planned or already initiated action. This *so-called* motor inhibition plays a crucial role in everyday life. In life-threatening situations such as holding yourself from crossing the street when noticing a fast approaching car, efficient inhibitory control is even key to survival. Despite the fact that observing or measuring inhibition may be challenging as it implies absence of behavior, clearly, the role of inhibitory motor control should not be underestimated as it is of key importance for adaptability in many real-world situations. Clinical expressions of deficits in inhibitory function illustrate this such as for example Tourette Syndrome where inhibitory failure leads to production of awkward movements or sounds. Attention-Deficit/Hyperactivity Disorder also illustrates how lack of inhibition compromises everyday function and mental development. More recently, inhibition has also become a prominent focus of attention in aging research because there is mounting evidence that inhibitory motor function gradually declines with advancing age [1]. This does not only contribute to impaired motor control in general but also to general alterations in brain functioning, such as expansion of neural activity, reduced neural distinctiveness of neural representations, and increases in connectivity among the brain networks.

Interestingly, recent developments in brain-behavioral approaches have provided a new window into the neural mechanisms mediating efficient inhibitory control in the context of healthy aging [2]. This topic has been investigated from the cellular to the systems level. At the cellular level, γ-aminobutyric acid (GABA) is known to be the most prominent inhibitory neurotransmitter in the mammalian brain. At the systems level, one way to probe the GABA system in the human brain is the use of non-invasive brain stimulation techniques such as transcranial magnetic stimulation (TMS). This technique identifies intracortical inhibitory circuits in the primary motor cortex, as driven by GABA_A_ and GABA_B_ receptor activity. However, whereas TMS studies can only provide an indirect measure of inhibitory mechanisms and its *implicated* neurotransmitter, recent advances in medical imaging techniques allow scientists to accurately quantify the level of neurochemical compounds such as GABA in the brain by means of magnetic resonance spectroscopy (MRS) [3]. Furthermore, in comparison with TMS, MRS allows for the registration of GABA across the broader cortical/subcortical territory and thus reaches beyond the primary motor cortex.

To date, considerable research efforts have already been devoted to the study of the GABAergic system in the aging brain. In that regard, both animal and human research has shown age-related alterations in the GABA system, either related to (1) the synthesis of GABA at the presynaptic level, (2) altered subunit composition of both GABA_A_ and GABA_B_ receptors at the postsynaptic level, (3) a reduction of GABA level assessed with MRS, and (4) altered intracortical inhibitory circuits as measured with TMS [4,5]. These findings have sparked interest in using aging as a model for identifying the role of GABA in motor inhibitory control.

Recently, we used MRS to register baseline GABA levels within key regions of the motor inhibition network in order to identify its role in age-related deterioration of inhibitory control [2]. We acquired GABA levels in various cortical as well as subcortical regions constituting the motor inhibition network, i.e. the pre-supplementary motor area, right inferior frontal cortex, left sensorimotor cortex and bilateral striatum [6]. In line with previous work, our results indicated that older adults showed deficits in inhibitory control as determined with a task requiring suppression of a planned movement following presentation of a visual warning signal shortly before movement initiation. With respect to the neural process of inhibition, our MRS data indicated that older adults exhibited reduced levels of the neurochemical compound GABA within regions of the motor inhibition network as compared to their younger counterparts. More specifically, older adults with lower GABA levels within the pre-supplementary motor area were found to show poorer inhibitory motor control. Thus, the level of GABA in a key node of the inhibition network was predictive for efficient inhibitory control at the behavioral level.

There is mounting evidence that the integrity of the GABA system is critically important for efficient motor inhibition. More specifically, the registration of brain GABA levels within the motor inhibition network provides a window into the mechanisms mediating age-related declines in inhibitory motor control. But GABA is not only critical for inhibitory control. Modulation of GABA is important for inducing processes of neuroplasticity that enable the shaping of our actions and the generation of new behaviors. In view of the demographic evolution of society, the importance of research on age-related alterations in the GABA system cannot be underestimated. This work may contribute to a body of knowledge that may lead to the development of interventions targeting the GABA system with the ultimate goal to ameliorate age-related declines in motor inhibition.
